# The Protective Effect of Lavender Essential Oil and Its Main Component Linalool against the Cognitive Deficits Induced by D-Galactose and Aluminum Trichloride in Mice

**DOI:** 10.1155/2017/7426538

**Published:** 2017-04-26

**Authors:** Pan Xu, Kezhu Wang, Cong Lu, Liming Dong, Li Gao, Ming Yan, Silafu Aibai, Yanyan Yang, Xinmin Liu

**Affiliations:** ^1^Research Center of Pharmacology and Toxicology, Institute of Medicinal Plant Development (IMPLAD), Chinese Academy of Medical Sciences and Peking Union Medical College, Beijing 100193, China; ^2^Department of Pharmacology and Toxicology Laboratory, Xinjiang Institute of Traditional Uighur Medicine, Urumqi, Xinjiang 830049, China; ^3^China Astronauts Research and Training Center, Beijing 100094, China

## Abstract

Lavender essential oil (LO) is a traditional medicine used for the treatment of Alzheimer's disease (AD). It was extracted from* Lavandula angustifolia* Mill. This study was designed to investigate the effects of lavender essential oil (LO) and its active component, linalool (LI), against cognitive impairment induced by D-galactose (D-gal) and AlCl_3_ in mice and to explore the related mechanisms. Our results revealed that LO (100 mg/kg) or LI (100 mg/kg) significantly protected the cognitive impairments as assessed by the Morris water maze test and step-though test. The mechanisms study demonstrated that LO and LI significantly protected the decreased activity of superoxide dismutase (SOD), glutathione peroxidase (GPX), and protected the increased activity of acetylcholinesterase (AChE) and content of malondialdehyde (MDA). Besides, they protected the suppressed nuclear factor-erythroid 2-related factor 2 (Nrf2) and heme oxygenase-1 (HO-1) expression significantly. Moreover, the decreased expression of synapse plasticity-related proteins, calcium-calmodulin-dependent protein kinase II (CaMKII), p-CaMKII, brain-derived neurotrophic factor (BDNF), and TrkB in the hippocampus were increased with drug treatment. In conclusion, LO and its active component LI have protected the oxidative stress, activity of cholinergic function and expression of proteins of Nrf2/HO-1 pathway, and synaptic plasticity. It suggest that LO, especially LI, could be a potential agent for improving cognitive impairment in AD.

## 1. Introduction

Alzheimer's disease (AD) is an age related neurodegenerative disease of the brain that is the most common cause of dementia in the world. The disease is characterized by a progressive deterioration of cognitive functions [1]. Learning and memory process is linked with the induction of long-time potential, which is interpreted as the retention of information. LTP could ensure long-term changes in synaptic efficacy in distributed networks, leading to persistent changes in the behavioral patterns, actions, and choices [2]. The hippocampus is necessary for LTP, in which the changed level of synaptic protein, neural substrates, and neurotransmitters in AD might directly lead to the loss of memory formation, storage, or retrieval [3]. The cognitive damage interferes with the normal life of patients and places a considerable burden on society. Recent studies showed that combined administration of chronic D-gal and AlCl_3_ has induced obvious cognitive impairments [4–6]. Even before the cognitive changes appear, the pathological changes, including abnormal cholinergic system, mitochondrial dysfunction, apoptosis, amyloid-*β* peptide (A*β*) deposition, and especially obvious oxidative stress, were observed in the model [6–9]. It suggested that the D-gal and AlCl_3_ induced model is a useful model for the drugs screening in AD.

Though the exact mechanism of AD remains unclear, now oxidative stress has been proposed to be a critical factor in the course of AD [10]. The imbalance between the production of reactive oxygen species (ROS) and antioxidant defense systems could cause oxidation of biological macromolecules, aggravating mitochondrial dysfunction and ultimately leading to cell death or neurodegeneration [11]. The hippocampus is vulnerable to that oxidative damage [12]. There are many endogenous antioxidant enzymes and heme oxygenase-1 (HO-1) is one of the most effective [13]. Pharmacological induction of HO-1 could give an adaptive survival response against oxidative insults [14]. Nuclear factor-erythroid 2-related factor 2 (Nrf2) is an upstream transcription factor, regulating HO-1 expression [15]. Thus, antioxidant therapy, especially those based on Nrf-2 and HO-1 targets, might play an important role in the prevention and treatment of AD.

Lavender, which is used for therapeutic and cosmetic, belongs to the family Labiatae (Lamiaceae) and species* Lavandula angustifolia* Mill.. Traditionally, Lavender has been used to treat anxiety, insomnia, depression, convulsion, pain, and cerebrovascular diseases in many countries [16]. Lavender essential oil (LO), isolated from Lavandula plants, is the most common used form of lavender. LO was reported to possess various biological activities in central nervous system and show effectiveness in the management of depression, anxiety, stress, and cerebral ischemia [17–20]. Recent studies found that LO has neuroprotective and cognitive improvement effects in some AD models though antioxidant properties [21, 22]. (−)-Linalool (LI), as the major component of LO (40%), is also responsible for its therapeutic properties [23]. LI has antioxidant, anti-inflammatory, antitumor, antidepressant, and antimicrobial activities. Increasing evidences demonstrated that the antioxidant activity of LI could protect neurons from neurotoxicity and damage. Moreover, LI protected neuropathological and behavioral impairments in old triple transgenic AD mice [24].

Though there were some previous studies about the potential protective effect of LO or LI on the cognitive and pathological impairments in AD models, their pharmacological study in D-gal and AlCl_3_ induced mice, an excellent AD model, was seldom reported and the related mechanism research was limited. Therefore, the present study investigated the effects of LO and LI on cognitive deficits induced by D-gal and AlCl_3_ in mice using Morris water maze and step-though tests. Meantime, the changes of oxidative stress in mice were also analyzed. Additionally, we detected Nrf2, HO-1, calcium-calmodulin-dependent protein kinase II (CaMKII), p-CaMKII, BDNF, TrkB expression, and AChE activity to explore the possible action mechanisms of LO and LI.

## 2. Materials and Methods

### 2.1. Drugs and Materials

**Figure 1:**
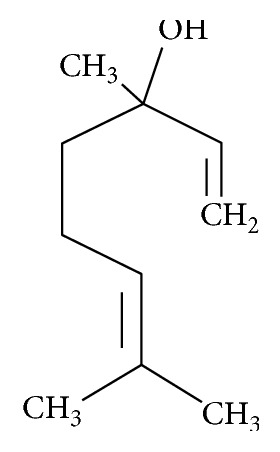
Molecular structure of linalool.

The lavender essential oil (LO) was provided by Yili Brother Biotech, Inc (Xinjiang, China). LO is extracted by steam distillation from* Lavandula angustifolia* grown in Xinjiang and harvested in August 2015. Linalool (LI, Figure 1) with a purity of 98.5% determined by HPLC was obtained from the National institutes for Food and Drug Control (ID: 4BJH-UDPM, Beijing, China). D-galactose (D-gal) and aluminum trichloride (AlCl_3_) were purchased from (Amresco Company, Solon, OH, USA). Assay kits of superoxide dismutase (SOD), glutathione peroxidase (GPX), malondialdehyde (MDA), and acetylcholinesterase (AChE) were purchased from Nanjing Jiancheng Biotechnology Institute (Nanjing, China). Rabbit antibodies to HO-1 and Nrf2 (1 : 1000, Abcam, USA) were form Abcam (Cambridge, MA, USA) and rabbit antibodies to CaMKII, p-CaMKII, BDNF, TrkB, *β*-actin, and Horseradish peroxidase- (HRP-) conjugated goat anti-rabbit IgG secondary antibodies were from Cell Signaling Technology (Boston, MA, USA). Protein extraction kit and BCA protein assay kit and ECL kit were from Cwbio (Beijing, China).

### 2.2. Lavender Oil Chromatographic Analysis

The chemical composition of LO was confirmed and analyzed using Gas Chromatography-Mass Spectrometer (GC-MS, Thermo Fisher Trace 1310/ISQ Mass Selective detector), with a column of Agilent HP-5MS capillary column (30 m × 0.25 mm × 0.25 *μ*m). The sample was injected by splitting (split ratio, 1 : 100). The oven temperature was programed as follows: 50°C, 1 min; 2°C/min to 100°C for 5 min; 2°C/min to 240°C for 5 min. The injector and detector temperatures were 250°C and 280°C, respectively. Carrier gas was helium at a flow rate of 1 mL/min. Mass range was 20–400* m/z*. The volatile compounds were identified by comparing their retention indices (RI) or mass spectra with those of reference substances, NIST/NBS libraries and literature data. Relative abundances of the components were derived by using the same instrumentation with the flame ionization detector (FID) configuration. Data extraction and analysis were performed using the Thermo Xcalibur software. The constituents were expressed as percentages from peak area normalization, assuming that the total injection was 100% of essential oil.

### 2.3. Animals and Drug Administration

A total of 90 male C57BL/6J mice (12 weeks) were purchased from the Vital River Laboratories (Qualified No.: SCXK 2012-0001, Beijing, China). They were housed under temperature controlled condition (25°C) with alternating light/dark cycle (lights, 8:00 AM–8:00 PM) and were given a standard diet and water ad libitum. Mice were allowed to acclimatize for 1 week before the beginning of the experiment. All experimental procedures were carried out under the approval and supervision of the Academy of Experimental Animal Center of the Institute of Medicinal Plant Development and in accordance with the NIH Guide for the Care and Use of Laboratory Animals.

The mice were randomly divided into 6 groups, control (given vehicle), AD group (given vehicle), LO + AD groups (50, 100 mg/kg/d), and LI + AD groups (50, 100 mg/kg/d). The D-gal and AlCl_3_ were dissolved in sterile saline, while LO and LI were dissolved in vehicle that is a normal saline solution with 2% Tween-80 and 1% DMSO, respectively. Mice in AD and drug groups were injected intraperitoneally with D-gal (60 mg/kg/day) and AlCl_3_ (5 mg/kg/day) while the control group was treated with the same volume of sterile saline for 8 weeks. Then the behavioral tests were performed. From the fifth week of modeling to the end of behavioral tests (lasted 37 days) mice were given the corresponding drug solutions of their own groups. We observed that in the fourth week of drug treatment session, the administration area in mice abdominal skin was hardened with high dose LO treatment. This phenomenon did not appear in the other groups, which might suggest the skin irritation of high dose LO.

### 2.4. Behavioral Test

#### 2.4.1. Open-Field Test (OFT)

Locomotor activity was firstly assessed to preclude its interference in cognitive function parameters [25]. The open-field computer-aided controlling system consists of four metal boxes (30 × 30 cm, height 40 cm) with a 120 Lux light source on the ceiling, and a video camera fixed at the top. 30 minutes after administration, each mouse was placed at the center of the tank and allowed to adapt freely for 3 min. Then in the following 10 min, distances travelled were recorded automatically as the index.

#### 2.4.2. Morris Water Maze Test (MWM)

The MWM test was performed to evaluate the spatial learning and memory following OFT [26, 27]. The maze was a circular pool filled with water (24–26°C) which was opaque by adding milk. The pool was divided into four equal quadrants, one of which (e.g., SE) contains a hyaline platform (6 cm in diameter and 15 cm in height) submerged 1 cm below the surface. The swimming activity was recorded using a video camera overhead and analyzed via a computerized image analyzer system. In navigation experiment of 5 days containing four trial sessions per day, mice were released from one of four quadrants randomly and allowed to find the platform in 60 s. Before and after swimming, they were left on platform for 10 s. The escape latency and the escape rate were analyzed by a tracking and image analyzer system. Probing test was conducted with the platform removed 24 h after navigation. Mice were released from the quadrant (e.g., NW) opposite from the previous platform location (target quadrant) to receive 60 s memory retention test. The time in target quadrant and crossing number were analyzed.

#### 2.4.3. Passive Avoidance Task (PAT)

The passive avoidance test was performed one day after MWM test according to previous method [28]. The apparatus in trough-shape consisted of a white illuminated camber and a dark camber (17 cm × 13.5 cm × 25 cm, resp.). In training trial, following 180 s adaptation, each mouse was put into the light chamber to explore with the door opened for 300 s. When it entered the dark chamber, a 0.5 mA electric foot shock (5 s) was delivered. 24 h later, the consolidation trial was performed in the same way as training. Latency to enter the dark chamber and error time were recorded in 300 s test session.

### 2.5. Brain Sample Preparation

After the last behavioral test, all mice were anesthetized and decapitated quickly. Their brains were removed immediately on ice, then the hippocampus and the cortex were dissected out and frozen in liquid nitrogen. The samples were stored at −80°C for further determination.

### 2.6. Oxidative Parameter Assay in the Hippocampus and the Cortex

The hippocampi (*n* = 6) were weighted and sonicated with cold normal saline (1 : 10). Then the homogenate was centrifuged at 3,500 rpm/min (10 min, 4°C) and the supernatants were collected for assay. The SOD, GPX activities, and content of MDA were measured using commercially available assay kits according to the protocols, respectively. Briefly, the assay of SOD activity was based on its ability to inhibit the oxidation by superoxide anion free radical generated by the xanthine–xanthine oxidase system, thereby reducing the formation of water-soluble tetrazolium- (WST-) 1 formazan. The absorbance was read at 450 nm with a microplate reader. The GPX activity was assayed by measuring the decline of nicotinamide adenine dinucleotide phosphate reduced form (NADPH) in a coupled system at 340 nm, which was based on it catalyzing the oxidation of reduced glutathione to oxidized glutathione. The content of MDA was assayed at 535 nm by monitoring thiobarbituric acid reactive substance (TBARS) formation as previously described previously [29].

### 2.7. Determination of Acetylcholinesterase (AChE) Activity

AChE activity in the hippocampus and cortex were measured according to the protocol of assay kit. The prepared supernatant of the hippocampus and cortex was reacted with thiol agents to form trinitrobenzene at 37°C; then the reaction mixture was detected at 412 nm. AChE activity was expressed as U per mg of protein.

### 2.8. Western Blotting

Total proteins from hippocampus were isolated according to the instructions of the protein extraction kit. Briefly, the hippocampus was homogenized in ice-cold lysis buffer and then centrifuged for supernatant collection. The protein concentration was determined using a BCA protein assay kit. Protein lysates were separated by SDS-PAGE and transferred onto polyvinylidene difluoride (PVDF) membranes (Millipore Company, USA). After blocking in 5% nonfat dry milk-Tris buffered saline with Tween-20 (TBST) for 3 hours, the membranes were incubated overnight at 4°C with corresponding primary antibodies, including anti-HO-1 (1 : 1000), anti-Nrf2 (1 : 1000), anti-CaMKII (1 : 1000), anti-p-CaMKII (1 : 1000) anti-BDNF (1 : 1000), anti-TrkB (1 : 1000), and anti-*β*-actin (1 : 5000). After rinsing three times with TBST, membranes were incubated with a HRP-conjugated secondary antibody for 2 h at room temperature. Following postsecondary washes, the protein bands were visualized using an enhanced chemiluminescence (ECL) kit; then their intensities were scanned and analyzed with a Quantity One Software (Bio-Rad, Hercules, USA).

### 2.9. Statistical Analysis

Data were analyzed using the SPSS 17.0 software package (Chicago, IL, USA) and expressed as means ± standard error mean (SEM). Group differences in escape latency and escape rate during acquisition of MWM trials were analyzed using repeated-measure two-way ANOVA. The other data were analyzed by one-way ANOVA followed by Tukey's post hoc test to detect intergroup differences. The results of statistical analysis were performed with GraphPad Prism software 5.0 (GraphPad Software, CA, USA). *P* ≤ 0.05 was regarded as significant.

## 3. Results

### 3.1. Identification of LO Constituents

The chemical analysis of the lavender oil identified 31 constituents, and the following 20 constituents showed in Table 1 presented 91.31% of total LO composition. The main components of LO were linalool (37.96%) and linalyl acetate (29.34%), both of which accounted for 70% closely. The following were trans-*β*-Ocimene (8.6%), Terpinen-4-ol (3.54%), lavandulyl acetate (2.68%), (±)-Lavandulol (1.45%), eucalyptol (1.22%), and caryophyllene (1.1%). Some other components were also traced in LO.

### 3.2. Effect of LO and LI on Open-Field Test

**Figure 2:**
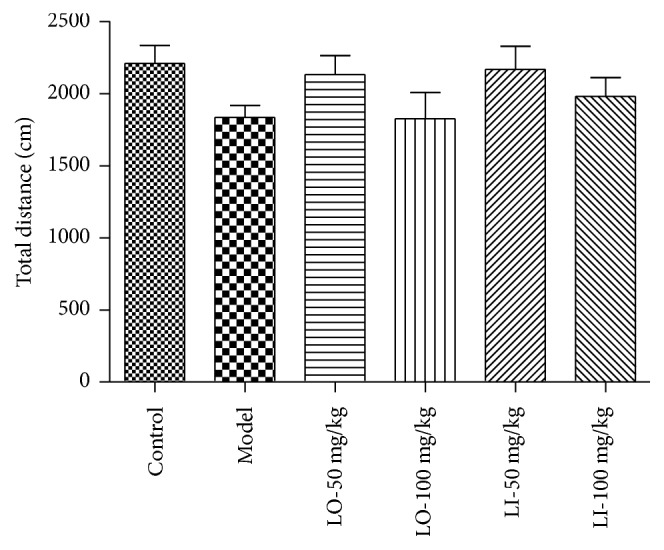
Effect of LO and LI on the total distance travelled in open-field test. Data are expressed as means ± SEM. *n* = 12-13 in each group.

The locomotor activity was evaluated by open-field test. As shown in Figure 2, there were no significant changes in total distance amongst all groups, though LO + AD (100 mg/kg) group and model group exhibited shorter total distance. The results excluded the influence of locomotor activity change on the evaluation of cognitive function.

### 3.3. Effect of LO and LI on MWM Test

**Figure 3:**
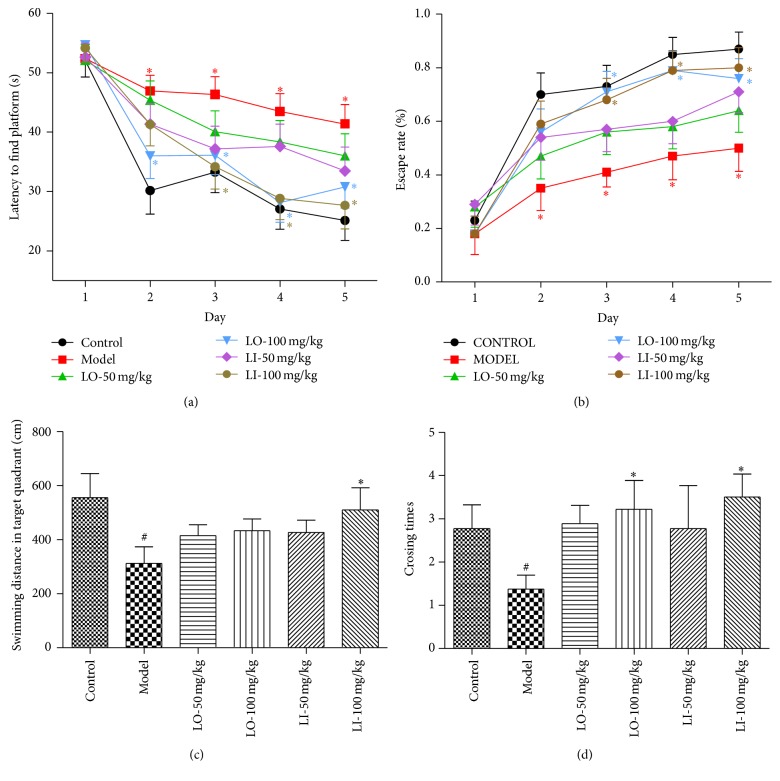
Effect of LO and LI on D-gal and AlCl_3_ induced cognitive deficits in MWM test. Latency to find platform (a) and escape rate (b) were measured for 5-day navigation. Swimming distance in the target quadrant (c) and crossing number (d) were recorded during the probe trial. Values are presented as mean ± SEM (*n* = 12 in each group). ^#^*P* < 0.05, compared with the control group, and ^*∗*^*P* < 0.05 compared with the D-gal and AlCl_3_ treated group.

In the navigation task, the cognitive performance was indicated by escape latency (Figure 3(a)) and escape rate (Figure 3(b)). Analysis of escape latency revealed significant difference between training days (*F* = 36.315, *P* < 0.01) and between groups (*F* = 14.2, *P* < 0.01). Further comparison demonstrated that the D-gal and AlCl_3_ treated mice had a longer escape latency than control mice from the second day (*P* < 0.5); LO (100 mg/kg) or LI (100 mg/kg) treatment could significantly shorten the latency prolongation from day 2 to day 5 (*P* < 0.5) or from day 3 to day 5 (*P* < 0.5). LI (50 mg/kg) treatment also showed effect in the third day (*P* < 0.5). Meanwhile, the main effects for day and group were significant in escape rate (*F* = 42.18, *P* < 0.01; *F* = 4.023, *P* < 0.05). Subsequent comparison showed the obvious difference of escape rate between the D-gal and AlCl_3_ treated mice and control mice from the third day (*P* < 0.05). With the treatment of LO (100 mg/kg) or LI (100 mg/kg), the low escape rate of model mice would be elevated significantly from the third to fifth day (*P* < 0.05).

Probing trial was preformed to evaluate the memory of the platform position. As shown in Figures 3(c) and 3(d), the distance in target quadrant (*P* < 0.05) and the crossing number (*P* < 0.05) in D-gal and AlCl_3_ treated mice were declined significantly compared with control group. However, the shorter distance in target quadrant in model group was reversed obviously by LI (100 mg/kg) (*P* < 0.05), while the crossing number was increased with LO (100 mg/kg) or LI (100 mg/kg) treatment (*P* < 0.05).

### 3.4. Effect of LO and LI on Step-Through Test

**Figure 4:**
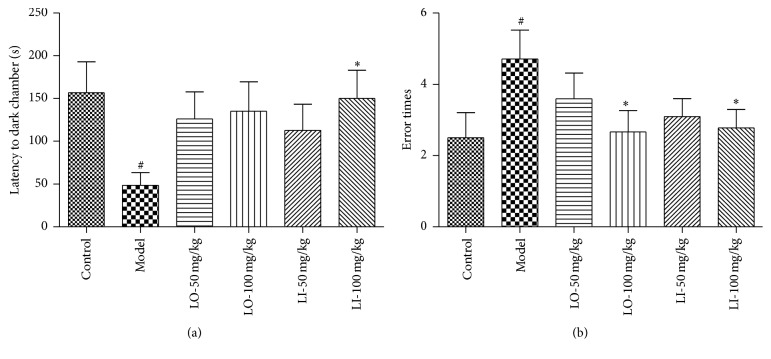
Effect of LO and LI on D-gal and AlCl_3_ induced cognitive deficit in step-through tests. Latency into dark chamber (a) and error times (b) were detected in consolidation trial. Data are shown as mean ± SEM (*n* = 12 in each group). ^#^*P* < 0.05 compared with the control group and ^*∗*^*P* < 0.05 compared with the D-gal and AlCl_3_ treated group.

In step-though test which could evaluate memory retention, the D-gal and AlCl_3_ treatment induced shorter latency into dark chamber and more error times compared with control group (*P* < 0.05). As the Figure 4 shown, LI (100 mg/kg) treatment prolonged the latency and reduced error times of model mice significantly (*P* < 0.05), while the treatment of LO (100 mg/kg) decreased error times close to the level of control group (*P* < 0.05).

### 3.5. Effect of LO and LI on Oxidative Stress Markers in the Hippocampus and the Cortex

As in Table 2, the activity of SOD and GPX was decreased significantly (*P* < 0.05) in hippocampus and cortex in groups treated with D-gal and AlCl_3_, which was protected significantly (*P* < 0.05) with the treatment of LO (100 mg/kg) or LI (100 mg/kg) except there was no significance change in the activity of GPX with the treatment of LO.

MDA is an indicator of oxidative damage, its level was significantly higher in the hippocampus of D-gal and AlCl_3_ treated group than control (*P* < 0.05, Table 2). LO (100 mg/kg) or LI (100 mg/kg) administration decreased the level of MDA significantly in hippocampus as compared to AD mice (*P* < 0.05). However, MDA levels in the cortex showed no difference amongst all groups, though there was an increase tendency of model group compared with the control.

### 3.6. Effect of LO and LI on AChE Activity in the Hippocampus and the Cortex

AChE is a marker of cholinergic system dysfunction in the brain. Results in Table 2 showed that AChE activity in the hippocampus and cortex of the D-gal and AlCl_3_ group was increased significantly compared with that of control group (*P* < 0.01, *P* < 0.05). The LO (100 mg/kg) and LI (100 mg/kg) protected the activity of AChE in the cortex of model mice (*P* < 0.05) markedly. Besides, LI (100 mg/kg) also significantly reversed the D-gal and AlCl_3_ induced AChE activity increase in the hippocampus (*P* < 0.05).

### 3.7. Effect of LO and LI on Nrf2/HO-1 Pathway Protein

**Figure 5:**
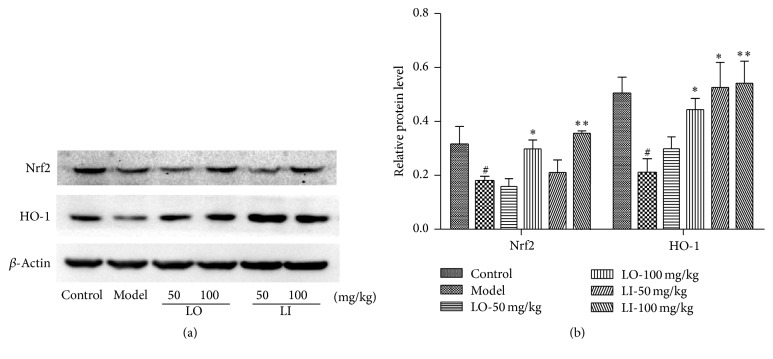
Effects of LO and LI on levels of Nrf2 and HO-1 in mice hippocampus. Mice were grouped and treated as described in the text. (a) The expressions of Nrf2 and HO-1 were measured by Western blot. (b) Quantitative analysis of bands density. The ratio to *β*-actin was calculated (*n* = 3 per group). Values are shown as mean ± SEM. ^#^*P* < 0.05 compared with control group and ^*∗*^*P* < 0.05 compared with D-gal and AlCl_3_ treated group.

The Nrf2/HO-1 signaling pathway participates in the protection against oxidative stress. Thus we investigated the expressions of Nrf2 and HO-1 of different groups using Western blot. As shown in Figure 5, D-gal and AlCl_3_ caused a (50%) decrease expression of Nrf2 and (60%) of HO-1 as compared to the level of control group. With the treatment of LI (100 mg/mL) or LO (100 mg/mL), the decreased levels of Nrf2 and HO-1 in model mice were protected significantly (*P* < 0.01, *P* < 0.05). Meanwhile, the lower dose of LI (50 mg/mL) increased the HO-1 expression obviously compared with model mice.

### 3.8. Effects of LO and LI on the Expression of Synapse Plasticity-Related Proteins in the Hippocampus

**Figure 6:**
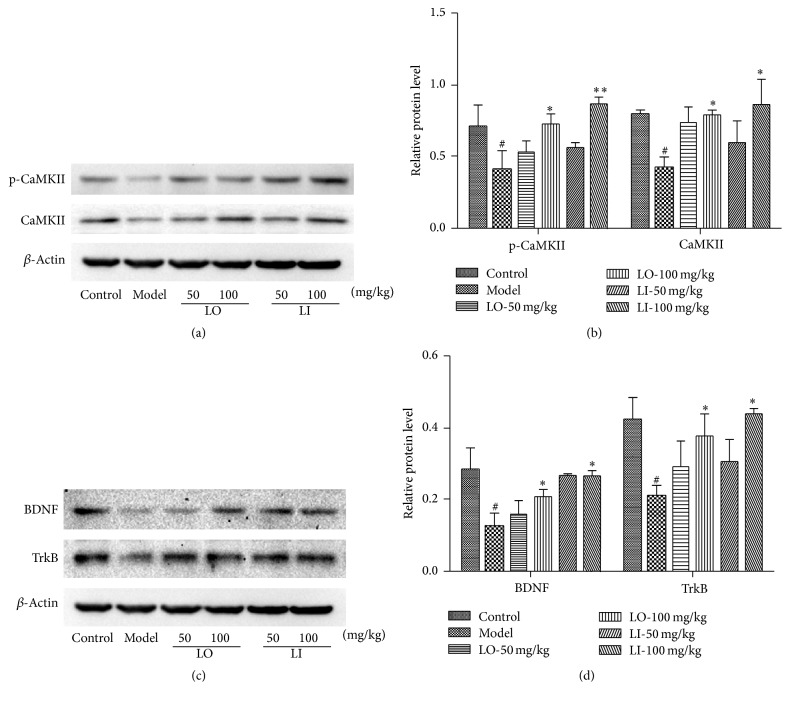
Effects of LO and LI on levels of BDNF and TrkB in mice hippocampus. Mice were grouped and treated as described in the text. The expressions of (a) CaMKII and p-CaMKII and (c) BDNF and TrkB were measured by Western blot. (b) and (d) Quantitative analysis of bands density. The ratio to *β*-actin was calculated (*n* = 3 per group). Values are shown as mean ± SEM. ^#^*P* < 0.05 compared with control group and ^*∗*^*P* < 0.05 compared with D-gal and AlCl_3_ treated group.

CaMKII is the key protein at the postsynaptic density necessary for synaptic plasticity. We investigated the effect of drugs on the expression of CaMKII and its phosphorylation by Western blot. Figures 6(a) and 6(b) showed that the D-gal and AlCl_3_ group had lower level of CaMKII and p-CaMKII than the control (*P* < 0.05). However, we found a significant increase in CaMKII and p-CaMKII expression in LI + AD (100 mg/mL) group as compared to AD group (*P* < 0.01, *P* < 0.05). Meanwhile, those two proteins levels in model group were also reversed with the LO (100 mg/mL) treatment significantly (*P* < 0.05).

BDNF, which binds to TrkB, has multiple and distinct functions in synaptic plasticity in brain [30]. The BDNF and TrkB expressions were evaluated and showed in Figures 6(c) and 6(d). Significant lower expression of BDNF and TrkB was observed in the hippocampus of D-gal and AlCl_3_ treated group when compared with the control (*P* < 0.05). Mice treated with LI (100 mg/mL) or LO (100 mg/mL) exhibited a significant increase in the expression of these proteins (*P* < 0.05).

## 4. Discussion

AD is a neurodegenerative disease and its prevalence is rising. The majority of AD cases are onset after the age of 65, accounting for more than 95% of the affected. D-gal is a reducing sugar in animals [31] which could be oxidized into hydrogen peroxide (H_2_O_2_) and aldehydes when intracellular level of D-gal was excess [32]. Long-term administration of D-gal induces behavioral and neurobiological changes similar to natural aging [33]. Aluminum owns neurotoxicity, which accelerates the pathogenic course of AD [34]. It was reported that D-gal and AlCl_3_ induced model mimic well the cognitive impairment and pathological changes in clinical AD [6]. Therefore, the D-gal and AlCl_3_ combined administration is a good model for AD study and drug screening and was used in our study.

Lavender essential oil is a well-known mixture mostly extracted from* L angustifolia*. LO and its major component, linalool, have shown multiple bioactivities especially for AD diseases [21, 24]. Since the composition of LO may be varies due to different origin, we examined the major contents of LO using GC-MS [35]. A total of 31 major constituents of the LO were identified and LI accounted for 37.96% as the main component and was similar to previous reports [36, 37]. Based on these results, we further investigated the cognitive effect of LO and LI in the D-gal and AlCl_3_ treated mice and its related mechanism.

In the behavioral study, chronic D-gal and AlCl_3_ administration led to the bad performance of mice in the Morris water maze test and step-though test, which was consistent with previous reports [38, 39]. Morris water maze tasks could reflect spatial memory ability of animal sensitively. The mice treated with LO or LI (100 mg/kg) have shown better cognitive function in navigation and probing process compared to the AD mice. Meanwhile, LO or LI (100 mg/kg) effectively reversed the D-gal and AlCl_3_ induced cognitive impairments in step-though test, which reflects the ability of retaining and recalling information [40]. Considering the fact that there was no difference in locomotor activity amongst groups, it suggested that the cognitive amelioration of drugs is likely to be mnemonic in origin, rather not provoked by sensorimotor effects. It is worth noting that at the same dose the cognitive improvement effect of LI tended to be more potent than LO pharmacologically. For example, swimming distance in target quadrant exhibited significant increase with LI treatment only. At a given concentration, LI in LO is diluted by the presence of the other components. It may partly explain the lower effect potency of LO compared with LI.

Oxidative stress is a key factor in the progression of AD, which can eventually induce cellular apoptosis in brain and cognitive damage [41]. ROS overproduction is widely observed in AD, which can be indicated indirectly by analyzing MDA, a product of lipid peroxidation. The activation of antioxidant enzymes, such as SOD and GPX, could scavenge ROS and protect cells against oxidative damage [42]. In our study, treatment with LO or LI (100 mg/kg) significantly protected the decreased activities of SOD and GPX induced by D-gal and AlCl_3_ in hippocampus and elevated level of MDA in AD mice. The results indicated that LO and LI own obvious neuroprotective effect against oxidative stress, which is correlated with the previous studies [21, 43]. It is likely the mechanism underlying their cognitive enhancement effect in behavioral tests.

In the antioxidation process, Nrf2, a transcription factor, is a major protective regulator. Nrf2 could be activated and bound with antioxidant response element (ARE), then inducing the expression of defensive genes, including HO-1 [44]. HO-1 and the products of heme degradation catalyzed by HO-1 are potent and sensitive antioxidant enzymes [13]. Besides, HO-1 could induce the expression of other antioxidant enzymes, such as SOD [45]. Our results showed that the expression inhibitory of HO-1 and Nrf2 induced by D-gal and AlCl_3_ administration was reversed markedly with LI or LO treatment. It suggested that neuroprotective effect against oxidative stress of LI or LO might be mediated by the Nrf2/HO-1 pathway.

The degeneration of cholinergic system in hippocampus and cortex is thought to be closely related to the cognitive deficits in AD [46]. AChE is responsible for hydrolysis of acetylcholine, which is vital for cognitive functions. Its activity is influenced by the alteration in the lipid membrane vulnerable to oxidative stress. AChE activity was increased in D-gal or aluminum treated animal brain [6, 47], which agreed with our result. With LO or LI treatment, the elevation of AChE activity in the hippocampus and cortex was prevented effectively, resulting in increased acetylcholine. Thus, normalization of AChE activity may be another mechanism underlying the cognitive improvement effect of LO and LI.

Accumulating evidence indicated that the initial decline of memory function in AD is due to changes in synaptic function [48]. Synaptic plasticity underlies learning and memory progress could be elucidated by long-term potentiation (LTP) of synaptic activity well. CaMKII activity is essential for normal N-methyl-D-aspartic acid (NMDA) receptor-dependent forms of LTP and spatial learning and memory. Induced by calcium elevation, CaMKII will become its autophosphorylation form, which will be translocated to combine with Ca-CaM complex. Their combination could regulate different postsynaptic proteins [49]. The levels of p-CaMKII were observed significantly reduced in AD models [50]. BDNF, a central neurotrophic factor binding to tyrosine kinase TrkB, promotes the various neurons survival, regulates axonal and dendritic growth, and enhances synaptic plasticity in the central nervous system [51]. It was documented that BDNF protein or mRNA are significantly reduced in the brain of AD patients [52]. In current study, the expressions of CaMKII, p-CaMKII, BDNF, and TrkB in the hippocampus were decreased significantly in D-gal and AlCl_3_ treated mice but were elevated by the treatment of LI or LO treatment effectively. The protected expression of those proteins is likely to induce the postsynaptic proteins of normal level, making sure of axonal and dendritic growth and the successful information of LTP. These results suggested that the cognitive effect of LI and LO may be partly due to its ability to enhance synaptic plasticity.

## 5. Conclusion

In summary, our study identified the composition of LO and demonstrated that LO and its main component LI significantly improved cognitive impairment induced by D-gal and AlCl_3_ in mice. The results indicated that these effects were related to alleviation of oxidative stress depending on Nrf2/HO-1 pathway, reversing the activity of AChE and enhancing the weakened synaptic plasticity. Taken together, it is suggested that LO, especially its main component LI, may have the potential to be developed as the drug for preventing or improving cognitive deficits in AD.

## Figures and Tables

**Table 1:** Chemical compositions of *Lavandula angustifolia* essential oil.

Peak number	Name of the constituents	Retention time (min)	Area (%)
1	*β*-Pinene	7.88	0.14
2	Propionic acid	8.1	0.77
3	1-Octyl-3-alcohol	8.87	0.28
4	Ethyl acetate	9.31	0.32
5	D-Limonene	9.92	0.31
6	Eucalyptol	10.08	1.22
7	trans-*β*-Ocimene	10.34	8.6
8	3-Carene	10.86	0.79
9	cis-Linaloloxide	12.1	0.12
10	Linalool	13.89	37.96
11	1-Octyl-3-alcohol acetate	14.35	0.49
12	(+)-2-Bornanone	16.64	0.13
13	(±)-Lavandulol	17.66	1.45
14	(+)-Borneol	18.16	0.56
15	Terpinen-4-ol	18.63	3.54
16	*α*-Terpineol	19.69	0.82
17	Linalyl acetate	22.95	29.34
18	Lavandulyl acetate	25.21	2.68
19	Caryophyllene	34.84	1.1
20	trans-*β*-Farnesene	38.18	0.69

**Table 2:** Effects of LO and LI on the activities of SOD, GPX, and AChE and the content of MDA in the hippocampus and cortex of D-gal and AlCl_3_ treated mice. Values are expressed as mean ± SEM (*n* = 10–12 in each group). ^#^*P* < 0.05, ^##^*P* < 0.01 compared with control group, and ^*∗*^*P* < 0.05 compared with AD group.

Groups		SOD	GPX	MDA	AChE
Control	Hip	201.64 ± 16.24	129.42 ± 8.39	79.20 ± 6.07	6.81 ± 0.26
	Cort	308.21 ± 45.21	181.14 ± 9.39	91.85 ± 1.93	7.33 ± 0.45
Model	Hip	161.98 ± 24.20^#^	98.86 ± 9.54^#^	100.40 ± 6.05^#^	9.42 ± 0.32^##^
	Cort	204.65 ± 26.11^#^	127.44 ± 15.56^#^	111.54 ± 5.64	9.21 ± 0.37^#^
LO-50 mg/kg	Hip	173.67 ± 29.06	114.88 ± 12.13	91.82 ± 7.70	9.06 ± 1.03
	Cort	228.07 ± 36.26	136.03 ± 19.86	105.02 ± 3.66	8.75 ± 0.23
LO-100 mg/kg	Hip	190.99 ± 12.67^*∗*^	127.87 ± 4.57^*∗*^	82.67 ± 6.59^*∗*^	8.06 ± 0.20
	Cort	292.86 ± 50.65^*∗*^	166.97 ± 6.54	98.33 ± 7.30	7.53 ± 0.59^*∗*^
LI-50 mg/kg	Hip	182.94 ± 11.76	109.61 ± 4.44	92.47 ± 4.77	8.85 ± 0.24
	Cort	270.18 ± 40.31	147.65 ± 14.32	101.35 ± 8.65	8.95 ± 0.62
LI-100 mg/kg	Hip	197.27 ± 7.46^*∗*^	128.90 ± 9.42^*∗*^	80.47 ± 3.36^*∗*^	7.96 ± 0.25^*∗*^
	Cort	302.52 ± 40.75^*∗*^	177.34 ± 14.33^*∗*^	93.67 ± 15.05	7.43 ± 0.44^*∗*^
